# Impact of linear programming-based optimization of pediatric hospital location and quantity on patient travel time in Germany

**DOI:** 10.1186/s12913-026-14042-y

**Published:** 2026-01-23

**Authors:** Dariusz Lesniowski, Nicolas Terliesner

**Affiliations:** 1 Charité – Universitätsmedizin Berlin, corporate member of Freie Universität Berlin and Humboldt-Universität zu Berlin, Department of Pediatric Pulmonology, Immunology and Intensive Care Medicine, Berlin, Germany; 2 Data Science Institute, Berlin, Germany; 3 Cardiac Intensive Care Unit, Great Ormond Street Hospital for Children NHS Foundation Trust, London, UK

**Keywords:** Geographical accessibility, Travel time optimization for healthcare services, Optimization, *p*-median problem, Healthcare accessibility

## Abstract

**Background:**

Access to pediatric medical care is a critical factor in determining health outcomes. Hospital landscape restructuring processes need to consider the geographical accessibility of pediatric emergency and inpatient services. Public regulators in Germany aim for a travel time to the closest pediatric emergency hospital below a threshold of 40 min. This study investigates whether optimizing the allocation of pediatric hospital services to existing hospital sites by different approaches can improve accessibility by reducing travel time and enhancing threshold compliance.

**Methods:**

We analyzed patient travel time by car to the nearest pediatric hospital using a comprehensive dataset of all hospitals in Germany, weighted by population density. Two optimization approaches were applied to minimize travel time by reallocating pediatric health services to existing hospital locations, *k*-means clustering and a *p*-median optimization method based on linear programming. We further evaluated the impact of adjusting the quantity of location-optimized pediatric hospitals on patient travel time.

**Results:**

Allocation optimization using linear programming reduced weighted median travel time by 5.5% (*p* < 0.0001), unweighted median travel time by 15.0% (*p* < 0.0001) and the proportion of patients with travel time exceeding 40 min by 36.1%. Travel time improvement using *k*-means clustering algorithm was less pronounced. Location optimization led to greater gains in compliance with the 40 minute threshold in rural than in urban areas. Quantity of hospitals was inversely associated with travel time length and proportion of patients with travel time above 40 min threshold. In a model that prioritized existing pediatric hospital locations, increasing the number of hospitals led to more marked travel time changes than decreasing hospital quantity.

**Conclusion:**

The study highlights the potential of linear programming as an approach for optimizing countrywide hospital service allocation to enhance geographical accessibility of specialized care. It emphasizes the necessity of considering optimization methods for restructuring healthcare infrastructure and provides a model that may facilitate planning processes at a supra-regional or countrywide scale.

**Supplementary information:**

The online version contains supplementary material available at 10.1186/s12913-026-14042-y.

## Introduction

The efficacy of medical services is influenced by a multitude of factors. Geographic accessibility is demonstrated to be a pivotal element, having an impact on the quality and the feasibility of treatment [1, 2]. Travel distance was shown to be related to the quality of medical care in adult patients, with patients residing farther from healthcare facilities experiencing poorer health outcomes. These include lower survival rates, longer hospital stays, and non-attendance at follow-up appointments [3]. Furthermore, the distance to medical facilities can also exert a negative financial impact on patients [4]. Although there is less evidence addressing pediatric care, geographic accessibility was demonstrated to also play a role in the quality of care provided to children [5–8]. With regards to Germany, the highest decision-making body in the German health care system (Gemeinsamer Bundesausschuss) has determined that the comprehensive coverage of departments for pediatric and adolescent medicine is at risk when patients under 18 years of age face a car travel time exceeding 40 minutes to reach the nearest emergency hospital. We demonstrated that in Germany, the mean travel time by car to the nearest acute hospital with pediatric inpatient services is shorter than 40 minutes. However, a part of the patients may face a travel time that exceeds this safety threshold [9].

Health care systems constantly undergo restructuring processes. Germany is currently facing political changes in the decision-making process of the distribution of hospital services. The number of hospitals in Germany decreased during the last decade and is likely to further decline. Consequently, there is a demand for models that support determining the optimal allocation of health services to the remaining hospitals, to ensure accessibility of health services. Most studies regarding the geographical accessibility of health services have focused on emerging healthcare systems where new infrastructure is being developed. However, these approaches may not fully apply to healthcare systems in industrialized countries like Germany, where the primary goal of resource planning may be to improve the efficiency of existing infrastructure or to consolidate and reduce the number of healthcare facilities. Therefore, this study focuses exclusively on optimizing the use of existing healthcare infrastructure. The objective of this study is to examine methods suitable for analyzing this real-world scenario and for providing guidance on the restructuring of the hospital landscape. It evaluates two different optimization approaches: a linear programming algorithm based on the *p*-median problem and a $k$-means clustering-based algorithm. Using Germany as a case study, this study aims to rearrange the distribution of pediatric emergency services to minimize patient travel time, based on the current hospital landscape and considering the potential for further consolidation of healthcare facilities.

## Materials and methods

### General considerations

Several methods have been proposed to optimize the geographical accessibility of healthcare services. The two-step floating catchment area method evaluates accessibility by considering both distance and the number of providers within a predefined catchment area [10]. However, it relies on fixed travelling limits and is more applicable to analyses on a regional than a countrywide level. The maximal coverage location problem maximizes the population covered within a set travel time or distance but neither aims at minimizing the overall travel burden nor does it address extremely low or high travel time [11]. In contrast, the *p*-median problem aims at minimizing the total travel burden. This method identifies the most efficient distribution of limited resources across predefined locations by minimizing overall travel distances or times between demand points and service facilities. It has been used to identify optimal hospital distributions with a reduced travel cost for the population, to evaluate existing travel burdens based on Euclidean distance [12]. Furthermore, it has been applied to identify locations for new hospitals with minimal restrictions on the choice of sites [13]. Current information technology enables the integration of realistic travel time calculations with the *p*-median optimization method, further improving its applicability to real-world scenarios. Whereas the *p*-median approach seeks a globally optimal allocation by jointly optimizing all demand points and service facilities, the $k$-means clustering algorithm partitions demand locations, such as population centers, into spatially coherent clusters. Thereby it minimizes the average travel burden within each cluster. A $k$-means approach was recently used in an optimization model aiming at minimizing the proportion of patients above a set travel time threshold in Finland [14]. Both methods - the linear programming algorithm derived from the *p*-median problem and the $k$-means clustering-based approach - offer appropriate frameworks for optimizing a population’s travel burden to specialized healthcare facilities distributed across predefined sites. Consequently, both approaches were evaluated within this case study.

### Data acquisition

In Germany, hospitals are legally obliged to report on their work and structures in annual reports [15]. We extracted data regarding hospital services from reports for the year 2021. For each hospital, the provision of health services was further manually evaluated based on the hospitals’ websites (evaluation of website data undertaken between 01/04/2024 and 30/04/2024). Where hospitals or hospital departments had been closed, other publicly available information, such as newspaper reports, was additionally considered. Hospitals were regarded as acute general hospitals if they provided both medical and general surgical emergency and inpatient services. Hospitals were regarded as pediatric hospitals if they provided a pediatric emergency unit as well as pediatric inpatient services. The data were then manually reviewed by two independent reviewers. Geo-coordinates were added. The list of hospitals in Germany included $n=1023$ hospitals with acute inpatient services, noted as $h \in H$. $n_{ped} = 315$ of these were considered as hospitals with acute pediatric emergency and inpatient services, noted $h_{ped} \in H_{ped} \subset H$. This list was used as a basis for all subsequent computations.

### Geographical modeling and analysis

To calculate travel time, we covered the whole map of Germany with hexagons, using the H3 Hexagonal Hierarchical Geospatial Indexing System. Developed by Uber Technologies, Inc., H3 was created to enhance the flexibility and efficiency of spatial data analysis across maps [16]. It enables users to easily adjust the resolution of the coverage, making it an ideal tool for detailed and dynamic geographical analyses. We used the centroids $c \in C$ of hexagons as geographical points that represent homes of patients seeking treatment. With resolution 6, H3 provides a coverage of Germany with $m=10911$ centroids $C$, and at resolution 7, it provides $m=76379$ centroids $C$. This allowed us to work with various resolutions depending on the intensity of the calculations. Resolution 6 was applied for selection of hospital locations using linear programming. Resolution 7 was applied for choice of hospital locations using $k$-means algorithm, computation of travel time and all statistical measures, such as mean, median, interquartile range (IQR), and the proportion of patients with a travel time exceeding 40 minutes.

Information about population density was extracted from EuroStat based on NUTS - Nomenclature of Territorial Units for Statistics, a hierarchical standard for the unique identification and classification of administrative divisions of countries within the European Union member states. We used NUTS level 3 to obtain the most detailed geographical distribution of population density $p_c$ for centroid $c$ located in the corresponding region. To assess possible disparities between urban and rural populations regarding access to pediatric health services, each region corresponding to $c$ was classified according to the Eurostat Typology for Urban-Rural Regions (DEGURBA). This typology categorizes NUTS-3 regions into three groups: predominantly urban, intermediate, and predominantly rural areas [17].

For calculating travel time by car between centroid $c$ and hospital $h$, we used openrouteservice. Openrouteservice, a service developed by the Heidelberg Institute for Geoinformation Technology based on OpenStreetMap, calculates travel time. Openrouteservice is an open-source project and allows researchers to deploy its service locally, which ensures fast computation [18]. We used OpenStreetMap for Germany, downloaded from Geofabrik on 01/04/2024, as the base map for Openrouteservice calculations [19]. We calculated travel time by car between the hospitals $H$ and centroids $C$, noted as $t_{c,h}$. In addition to the plain travel time $t_{c,h}$, we calculated the weighted travel time with regards to the population density $p_c$, to obtain the weighted travel time $w_{c, h} = p_c \cdot t_{c,h}$. The weighted travel time from each centroid to each hospital forms an $m \times n$ matrix $W$, with rows corresponding to hospitals and columns corresponding to centroids/patient homes. For statistical analysis of travel time from the given centroids to the nearest hospital (of the respective set of hospital locations), the shortest travel time was chosen for each centroid. To determine the proportion of patients with travel time longer than 40 min, we estimated the population of each H3 hexagon by multiplying its area (determined using the European Terrestrial Reference System 1989 Lambert Azimuthal Equal Area Europe [EPSG:3035]) with the local population density according to NUTS. We evaluated whether the distribution of travel time followed a normal distribution using the Shapiro-Wilk test. None of the distributions were found to be normally distributed. Consequently, we assessed the statistical significance of all pairwise comparisons using the Mann-Whitney U test.

### Geo-optimization methods

The objective of our optimization was to identify a configuration of $n_{ped}$ pediatric hospitals such that the cumulative travel time from each $h_{ped}$ to the nearest centroid $c$ across all points $C$ is minimized. We tried to answer a hypothetical scenario: If it were possible to reallocate pediatric emergency services to hospitals across Germany, what distribution of these services would result in the shortest overall travel time for patients? Reallocation of health services to an existing set of already functioning hospitals is more feasible than the construction of new hospitals. Furthermore, the quantity of hospitals in Germany has been decreasing, so focusing on optimizing existing infrastructure is particularly relevant. Therefore, in our model we restricted the allocation of health services to the given current geographical locations of hospitals in Germany.

As the first approach, we applied a $k$-means clustering algorithm to determine optimal locations for pediatric hospitals, using $k$-means from the Python library scikit-learn 1.5.2. In summary, the $k$-means algorithm groups observations or locations (in this case, population locations, i.e. centroids $C$) into $k$ clusters. The algorithm iteratively assigns each location to the nearest cluster center and updates the cluster center positions until the overall distance between the locations and their assigned cluster centers is minimized. In this study, the number of clusters corresponded to the number of hospitals to be selected. The resulting cluster centers represented aggregated population centers that captured the spatial distribution of the population across Germany. Following clustering of population locations, each cluster center was matched to its nearest hospital location based on travel time, thereby linking population clusters to existing healthcare facilities. Finally, we calculated population density-weighted and unweighted travel time for the new set of pediatric hospital locations. In more detail, we first transposed matrix $W$, representing weighted travel time between various locations and pediatric hospitals. This allowed us to use each hospital, now represented by columns in the transposed matrix, as a distinct feature for the $k$-means algorithm. By setting the number of clusters to $n_{ped}$, our objective was to identify the configuration of locations that would result in the shortest cumulative travel time from centroids $C$. As a second approach to determining the optimal distribution of pediatric hospital locations, we applied a *p*-median problem optimization based on linear programming using the Python library PuLP 2.9.0. In this context, a set of potential facility locations (e.g., hospitals) and a set of population locations (demand points) are considered. The objective of the *p*-median problem is to select *p* facilities such that the total travel burden - defined in this study as the sum of travel times between population locations $C$ and their assigned hospital - is minimized. Conceptually, the model assigns each population location to its nearest selected facility while ensuring that exactly *p* facilities are chosen from the set of available sites. This results in an allocation pattern that minimizes the average travel time for the entire population. In more detail, we first defined the problem: Let $x_{c, h} \in \{0, 1\}$ be a binary variable, with 1 if patient $c$ is treated at hospital $h$, otherwise 0. $y_{h} \in \{0, 1\}$ be another binary variable, indicating 1 if the hospital $h$ treats patients. $w_{c, h} \in W$, again, is a weighted travel time. Our linear programming model to minimize was defined as follows:

1 $$\begin{aligned}& \min z = \sum_{c \in C} \sum_{h \in H} x_{c,h} \cdot w_{c,h} \nonumber \\ & \textit{subject to:} \nonumber \\ & \sum_{h \in H} x_{c,h} = 1, \quad \forall c \in C \end{aligned}$$

2 $$ x_{c,h} \leq y_h, \quad \forall h \in H, c \in C $$

3 $$\begin{aligned}& \sum_{h \in H} y_h = n_{ped} = 315 \\& x_{c,h} \in \{0,1\}, \quad \forall c \in C, h \in H \nonumber \\ & y_h \in \{0,1\}, \quad \forall h \in H \nonumber\end{aligned}$$

The goal was to minimize the total travel time. Condition (1) ensures that each patient is assigned to exactly one hospital. Condition (2) links the assignment of patients to hospitals with the operational status of the hospitals: a patient $c$ can only be assigned to a hospital $h$ if this hospital is active in treating patients, as indicated by the binary variable $y_h$. This means that if a hospital is not a pediatric hospital ($y_h$ = 0), no patient can be assigned to it. Condition (3) ensures that exactly $315$ hospitals, which is the number of pediatric hospitals in Germany, are utilized in the treatment of patients. There is an option to replace constraint (2) with the Big $M$ notation $\sum_{c \in C} x_{c, h} \leq M y_h$ for efficiency reasons, as it can reduce memory usage for large problem sizes. However, it generally provides a weaker lower bound and may slow down computation in branch and cut algorithms [20, 21].

For the optimization process, we used the PULP_CBC_CMD solver. This solver uses the CBC (COIN-OR branch and cut) algorithm. It initially solves a simplified version of the problem using the Simplex method, an optimization method that models the problem as a simplex with multiple dimensions. Once an optimal solution for this simplified problem is found, the solver applies the Branch and Bound method to ensure that the final solution meets all necessary integer constraints, such as assigning patients to hospitals [22].

In the following steps, we investigated how travel time changes when the number of hospitals is increased or decreased from $315$. In other words, will the travel time metrics change if we build more hospitals or close some and then optimize their locations? To explore this, we modified condition (3) to use different hospital quantities.

We first optimized pediatric hospital location allowing for the assignment of a pediatric hospital status to any of the given set of hospitals $h \in H$. To further enhance the relevancy of our model for hospital planning purposes, in a second step we prioritized pediatric over non-pediatric hospital locations. This led to different scenarios depending on the desired quantity of pediatric hospitals $n_{ped}$, resulting in a more differentiated optimization. Existing pediatric hospitals were either retained or supplemented with additional locations. We considered the following scenarios: If $n_{ped} = 315$, the $315$ existing pediatric hospitals remained unchanged. No optimization was needed, as this number corresponded to the current quantity of pediatric hospitals in Germany. If $n_{ped} < 315$, the non-pediatric hospitals were excluded from the optimization process. In this case, the optimal locations within the group of the existing 315 pediatric hospitals were selected to match the number $n_{ped}$. If $n_{ped} > 315$, the $315$ existing pediatric hospitals were retained. The additional number of $n_{ped} - 315$ further locations from the group of non-pediatric general hospitals were selected by optimization. This additional optimization would allow an increase in the quantity of pediatric acute hospital locations without altering the existing locations of pediatric hospitals.

For formal purposes, these extended conditions can be translated into the following linear program:

$$\begin{aligned}& \min z = \sum_{c \in C} \sum_{h \in H} x_{c,h} \cdot w_{c,h} \nonumber \\ & \textit{subject to:} \nonumber \\ & \sum_{h \in H} x_{c,h} = 1, \quad \forall c \in C \nonumber \\ & x_{c,h} \leq y_h, \quad \forall h \in H, c \in C \nonumber \\ & \begin{cases} \text{if } n_{ped} = 315: & y_h = 1, \quad \forall h \in H_{ped}; \\ & y_h=0 \text{for } h \notin H_{ped} \\ \text{if } n_{ped} < 315: & \sum_{h \in H_{ped}} y_h = n_{ped}; \\ & y_h=0 \text{for } h \notin H_{ped} \quad \\ \text{if } n_{ped} > 315: & y_h = 1, \quad \forall h \in H_{ped}; \\ & \sum_{h \in H} y_h = n_{ped} \end{cases} \nonumber \\ & x_{c,h} \in \{0,1\}, \quad \forall c \in C, h \in H \nonumber \\ & y_h \in \{0,1\}, \quad \forall h \in H \nonumber \\ & H_{ped} \subset H \nonumber \\ & |H_{ped}| = 315, \text{existing pediatric hospitals} \nonumber\end{aligned}$$

## Results

### Current travel time to hospitals in Germany

**Fig. 1:**
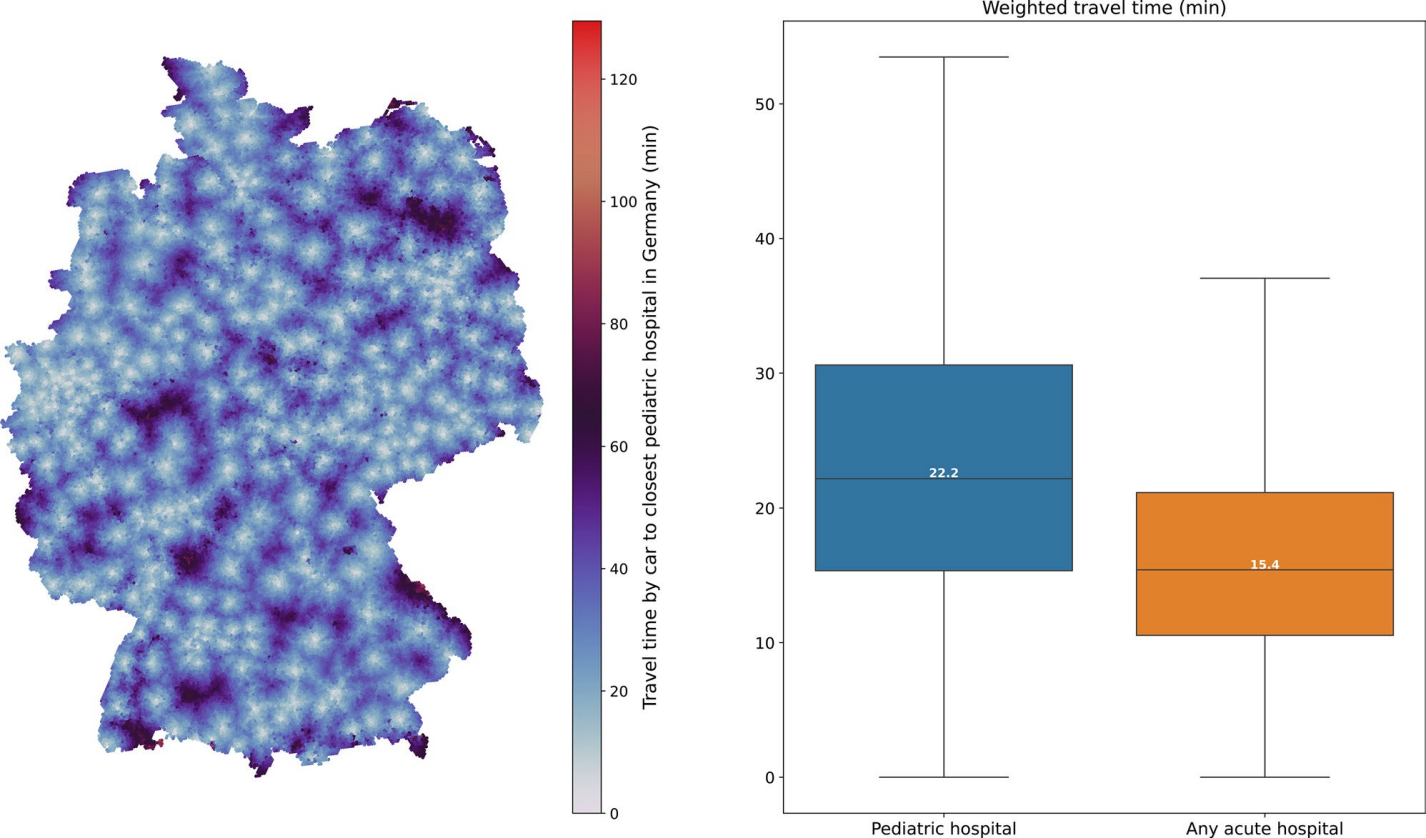
Travel time by car to closest hospital in Germany; left: travel time by car to closest pediatric hospital, mapped to geographical location of modeled patient homes; right: boxplots of weighted travel time (min) from modeled patient homes to nearest acute hospital (blue: pediatric hospital; orange: any acute hospital)

We first calculated the baseline travel time by car from the centroids to the current set of pediatric hospital locations in Germany (Fig. 1). The mean weighted travel time to any nearest acute hospital in Germany is 16.45 min (unweighted: 20.24 min). The mean weighted travel time to the nearest pediatric hospital is 23.79 min (unweighted: 29.39 min). The corresponding weighted median travel time is 15.42 min (IQR: 10.55–21.16 min) for any nearest acute hospital and 22.20 min (IQR: 15.35–30.61 min) for nearest pediatric hospital. Currently, 9.17% of pediatric patients are exposed to a travel time of more than 40 min to the closest pediatric hospital.

We then assessed regional disparities in travel time. The weighted mean travel time in predominantly rural regions is 32.00 min, compared to 26.64 min in intermediate regions and 18.84 min in urban regions (Table 2). The same pattern is observed for the weighted median travel time, which amounts to 31.47 min in rural areas, 25.59 min in intermediate areas, and 17.60 min in urban areas. Regarding the 40 minute travel time threshold, 2.11% of children in urban regions, 12.18% in intermediate regions, and 23.76% in predominantly rural regions exceed this limit. This corresponds to an estimated 2.7 million individuals residing in rural areas whose travel time to the nearest pediatric hospital exceeds 40 minutes.

### Travel time after optimization of pediatric hospital locations by $k$-means algorithm

Following optimization of pediatric hospital locations by $k$-means algorithm, the mean weighted travel time by car to the closest of 315 pediatric hospitals is 23.53 min (decrease by 0.26 min, compared to current weighted travel time; Table 1). The median weighted travel time by car is 22.39 min (increase by 0.19 min, compared to current weighted travel time; IQR: 16.34–29.63 min). Following optimization by $k$-means, the proportion of patients with a car travel time longer than 40 min is 6.42% (decrease by 2.75 percentage points, i.e. 30.00%, compared to the current proportion of patients).

**Table 1:** Comparison of weighted and unweighted metrics for different models; the mean, median, and interquartile range (IQR) are reported for both categories (min)

	weighted			unweighted		
model	mean	median	IQR	mean	median	IQR
current state	23.79	22.20	15.35 - 30.61	29.39	28.32	20.98 - 36.58
$k$-means	23.53	22.39	16.34 - 29.63	26.62	25.69	19.05 - 33.16
$p$-median	22.30	20.97	14.73 - 28.42	24.56	24.08	20.98 - 36.58

To assess the impact on access standards, we also analyzed the results of the *k*-means optimization by urban-rural region. The optimization led to the largest absolute changes in predominantly rural areas (Table 2). The weighted mean travel time in these regions decreased from 32.00 min (baseline) to 27.59 min. In predominantly rural regions, the proportion of the pediatric population with travel time exceeding 40 minutes decreased from 23.76% to 11.71%, corresponding to a relative reduction of 50.72% (12.05 percentage points). In intermediate regions, the proportion decreased from 12.18% to 9.00%, representing a relative reduction of 26.17% (3.18 percentage points). In predominantly urban regions, the weighted mean travel time increased slightly from 18.84 to 20.41 min, and the proportion of children exceeding the 40 minute threshold rose from 2.11% to 2.55%.

**Table 2:** Comparison of weighted travel time metrics (mean, median, and interquartile range (IQR) in minutes) to the nearest pediatric hospital across three regional typologies (Eurostat DEGURBA)

Region	Model	Mean	Median	IQR
Predominantly urban	Current State	18.84	17.60	12.60 - 23.70
	$k$-means	20.41	19.48	14.51 - 25.29
	$p$-median	18.41	17.05	12.23 - 23.15
Intermediate	Current State	26.64	25.59	18.58 - 33.44
	$k$-means	25.72	24.87	18.31 - 32.20
	$p$-median	24.63	23.75	17.49 - 30.33
Predominantly rural	Current State	32.00	31.47	23.66 - 39.51
	$k$-means	27.59	27.12	20.00 - 34.07
	$p$-median	28.50	28.06	20.55 - 35.38

### Travel time after optimization of pediatric hospital locations by linear programming

**Fig. 2:**
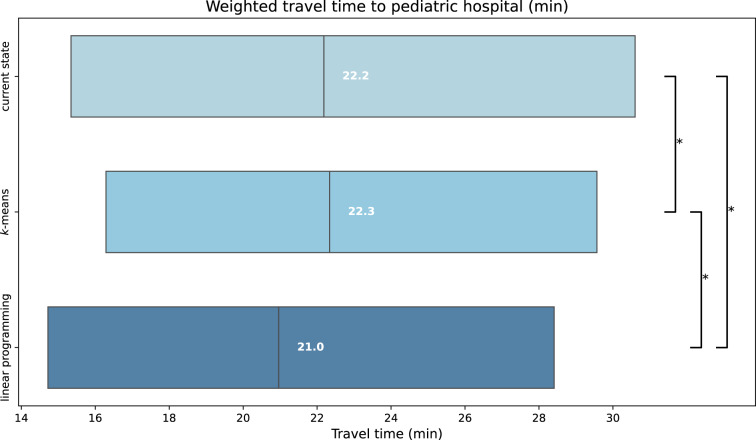
Boxplots of weighted travel time (min) from modeled patient homes to nearest of 315 pediatric hospitals; Upper: based on current pediatric hospital locations; Middle: after optimization by $k$-means clustering; Lower: after optimization by linear programming; * $p < 0.0001$

**Fig. 3:**
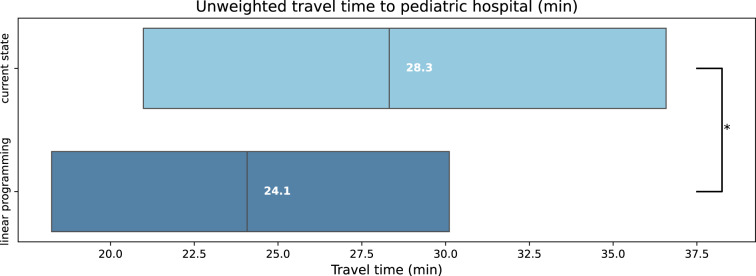
Boxplots of unweighted travel time (min) from modeled patient homes to nearest of 315 pediatric hospitals; Upper: based on current pediatric hospital locations; Lower: after optimization by linear programming;$^{*}$ $p < 0.0001$

Following optimization of the location of 315 pediatric hospitals by linear programming, the mean weighted travel time from modeled patient homes to the closest pediatric hospital by car is 22.30 min (decrease by 1.49 min, i.e. 6.3%, compared to current weighted travel time; Table 1). The median weighted travel time by car is 20.97 min (decrease by 1.23 min, i.e. 5.5%, compared to current weighted travel time; IQR: 14.73–28.42 min; $p < 0.0001$; Fig. 2). Following optimization by linear programming, the proportion of patients with a weighted travel time longer than 40 min to the closest pediatric hospital diminished to 5.86% (decrease by 3.31 percentage points, i.e. 36.1%). Unweighted travel time following optimization of pediatric hospital locations by linear programming shows a similar pattern as travel time weighted by population density, with a decrease in the mean travel time to the closest hospital by 16.4% (24.56 min after optimization, compared to 29.39 min current unweighted travel time). The median unweighted travel time decreased by 14.97% (−4.24 min; $p < 0.0001$; Fig. 3).

The separation of the linear programming results by region shows that the optimization reduced travel times across all urban-rural categories (Table 2). Following optimization by linear programming, the weighted mean travel time decreased to 18.41 min in urban regions, 24.63 min in intermediate regions, and 28.50 min in predominantly rural regions. In intermediate regions, the proportion of the pediatric population with travel times exceeding 40 min declined from 12.18% to 6.95%, corresponding to a relative reduction of 42.97% (5.23 percentage points). In predominantly rural regions, the proportion decreased from 23.76% to 14.44%, representing a relative reduction of 39.23% (9.32 percentage points). In predominantly urban regions, the proportion of children exceeding the 40 minute threshold increased slightly from 2.11% to 2.32% (0.21 percentage points).

### Impact of hospital quantity on travel time (liberal hospital selection)

**Fig. 4:**
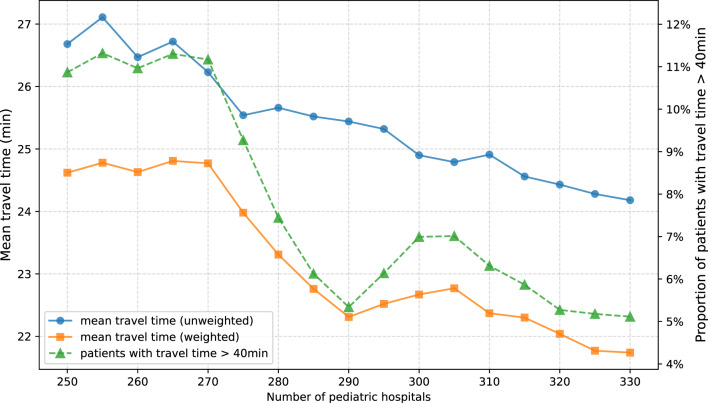
Travel time metrics based on the number of pediatric hospitals. Travel time was optimized using linear programming, allowing for any acute hospital location (including acute hospitals currently providing no pediatric services). Mean travel time from modeled patients’ homes to the nearest pediatric hospital (weighted and unweighted) and proportion of patients with travel times exceeding 40 min

Modifying the quantity of pediatric hospitals that are selected by optimization of hospital locations using linear programming affects mean car travel times from the modeled patient homes to the nearest pediatric hospital (Fig. 4). In the first model, allowing for liberal hospital selection regardless of whether the selected hospitals currently provide pediatric services, a decrease in the quantity of pediatric hospitals is generally associated with an increase in weighted and unweighted travel time by car. Conversely, an increase in pediatric hospital quantity is generally associated with a decrease in weighted and unweighted travel time. Increasing the number of pediatric hospitals by 15 (4.8% increase in hospital quantity) shortens the mean weighted travel time by 0.56 min (22.30 min vs. 21.74 min). Decreasing the number of pediatric hospitals by the same amount (15 hospitals) prolongs the mean weighted travel time by 0.93 min (1.7%). Mean unweighted travel time to the nearest pediatric hospital seems to keep an inverse linear correlation with hospital quantity throughout the analyses. On the contrary, decreasing the number of hospitals from 305 to 290 leads to a decrease in mean weighted travel time to the nearest pediatric hospital. A further decrease to 270 pediatric hospitals is related to a steep increase in travel time. When selecting less than 270 hospitals, travel time no further increases. While mean weighted (unweighted) travel time to the nearest pediatric hospital decreases by 11.7% (unweighted: 9.4%) when increasing the number of hospitals from 250 to 330, the proportion of patients with a travel time above 40 min decreases by 53.0%.

### Impact of hospital quantity on travel time (pediatric hospitals prioritized)

**Fig. 5:**
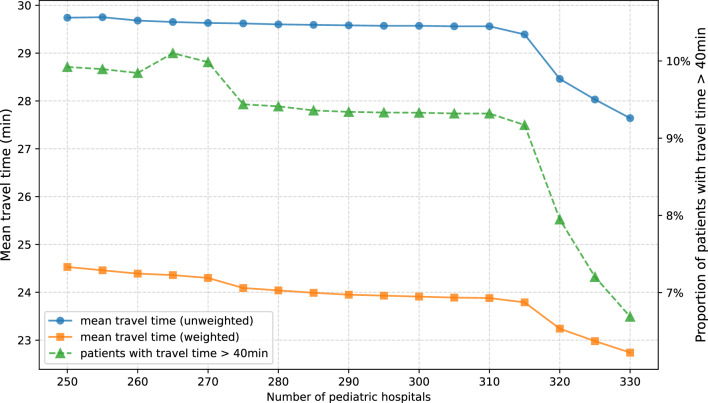
Travel time metrics based on the number of pediatric hospitals. Travel time was optimized using linear programming, restricting modeled hospital locations to current pediatric hospital locations where hospital quantity $n_{ped} \leq 315$ and adding currently non-pediatric hospitals as additional hospital locations where $n_{ped} > 315$. Mean travel time from modeled patients’ homes to the nearest pediatric hospital (weighted and unweighted) and proportion of patients with travel times exceeding 40 min

Modifying the quantity of hospitals, the prioritization of current pediatric hospital locations affected not only absolute mean travel time from patients’ homes to the nearest hospital, but also the relationship between travel time and the number of hospitals (Fig. 5). As with the previous model that did not prioritize current pediatric hospital locations, a decrease in hospital quantity is generally associated with an increase in mean (weighted and unweighted) travel time and in the proportion of patients travelling longer than 40 min to the nearest hospital. An increase in the number of pediatric hospitals is associated with a decrease in mean travel time and in the proportion of patients travelling longer than 40 min. In contrast to the prior, more liberal model, weighted travel time to the nearest pediatric hospital seems to demonstrate a linear association with the quantity of hospitals if $n_{ped} < 315$. Increasing the number of pediatric hospitals above 315, however, leads to a more marked decrease in mean weighted travel time (315 vs. 250 hospitals: 23.79 min vs. 24.53 min, i.e. increase by 0.74 min or 3.1%; 315 vs. 330 hospitals: 23.79 min vs. 22.74 min, i.e. decrease by 1.05 min or 4.4%). This also applies to the proportion of patients travelling longer than 40 min to the nearest hospital (315 vs. 250 hospitals: 9.17% vs. 9.92%, i.e. increase by 8.2%; 315 vs. 330 hospitals: 9.17% vs. 6.69%, i.e. reduction by 27.1%).

## Discussion

Our study investigated the potential to optimize the travel time to pediatric emergency health services on a countrywide level in Germany using a $k$-means clustering algorithm and a *p*-median optimization approach based on linear programming. Optimization with $k$-means clustering reduced the variance and the proportion of patients exceeding the 40 minute threshold but resulted in increased median travel time. The $p$-median approach using linear programming demonstrated a reduction in the proportion of patients travelling less than 40 minutes, as well as reduced mean and median travel time. When analyzing the results by urban-rural region, both optimization approaches showed the largest absolute and relative improvements in predominantly rural areas, where baseline travel times were highest. This indicates that these optimization models primarily affected regions with the poorest initial accessibility, which likely contributed to the substantial improvement in compliance with the 40 minute threshold. A model involving adjustment of the quantity of hospitals demonstrated that reducing the number of hospitals initially resulted in minor or no increase in travel time, with a sharper increase occurring beyond a critical reduction threshold. Conversely, increasing the number of hospitals consistently decreased travel time in linear programming models. In a model that prioritizes current pediatric hospital locations, the reduction of hospital quantity shows only a minor increase in travel time, compared with a greater decrease of travel time when adding further hospital locations.

Our findings regarding current mean travel time to pediatric emergency services based on H3 are in line with previous findings using randomly distributed geographical points to model patient homes in Germany [9]. Other studies have highlighted that clustering algorithms can improve access by redistributing patient catchment areas [14]. Optimization based on clustering may, as previously observed and confirmed in this study, minimize the number of patients who are at risk due to travel time above a set threshold. However, the increase in median travel time to the nearest pediatric emergency department observed with $k$-means clustering highlights the fact that optimizing for variance can inadvertently affect average metrics adversely. This study therefore does not support the use of $k$-means clustering based optimization of health service allocation to reduce patient travel time. Nevertheless, $k$-means clustering might serve as a valuable additional feature of a model and thus should still be considered for further investigation.

In contrast to $k$-means clustering, our study underlines the efficacy of the linear programming-based *p*-median approach in addressing multiple objectives related to health service allocation on a supra-regional level. This optimization algorithm may not only support the reduction of patients’ travel time to health care facilities but additionally prove to be especially effective in improving threshold compliance, i.e. minimizing the proportion of patients at risk due to long travel time.

Interestingly, in the model that does not prioritize current pediatric hospital locations, decreasing the number of pediatric hospitals from 305 to 290 results in a reduction in the mean weighted travel time to the nearest hospital. This counterintuitive finding may be explained by the optimization process, strategically relocating hospitals to more suitable locations. The calculations were based on travel time weighted by population density. The utilization of distance-based weights has the potential to accentuate the impact of hospital redistribution in densely populated regions, resulting in a net reduction in mean travel time. Given the discrete nature of hospitals, even small changes in hospital numbers can lead to shifts in regional accessibility patterns. Further reducing the hospital quantity from 290 to 270, however, leads to a sharp increase in mean travel time, as the system can no longer provide sufficient coverage for all regions. With fewer than 270 hospitals, mean weighted travel time does not increase further, suggesting a saturation effect where additional closures primarily impact patients in already underserved areas.

In the linear programming approach prioritizing current pediatric hospital locations, the model incorporates the non-optimal geographical distribution of existing pediatric emergency departments. With this background, interestingly, even ten percent of pediatric departments could be closed without major increases in mean travel time or the proportion of patients travelling more than 40 minutes. This might be attributable to a clustering of hospitals in congested urban areas, where closing of a single hospital could result in negligible effects. It indicates that the current distribution of services might hold a potential to close facilities at a minor cost of emergency health care access. Conversely, we observed marked travel time benefits by even slightly augmenting the quantity of pediatric hospitals. This may be attributable to a currently suboptimal service allocation. It demonstrates that even minor changes in the health service landscape, when based on a linear programming model such as the one this study proposes, may yield relevant benefit for travel time to health care services.

From a policy perspective, the findings primarily relate to future resource allocation and the planning of hospital landscape restructuring. By improving the efficiency of spatial service allocation, the optimization models may support a more balanced distribution of catchment areas and facilitate compliance with predefined access standards, such as the 40 minute travel time threshold. This could inform decisions on where hospital services might be consolidated without disproportionately affecting accessibility. Although the median travel time reduction of approximately 5–6% following optimization by linear programming may appear moderate at first glance, it should be interpreted in the context of the substantial improvement in threshold compliance. The proportion of the pediatric population exceeding the 40 minute benchmark decreased by more than one third, indicating a meaningful enhancement for those previously underserved. These findings suggest that optimization-based planning could be particularly relevant where hospital restructuring is already under consideration or where resources are being reallocated. The potential cost implications depend on the specific characteristics of the healthcare system and require further, case-specific economic analyses. While the results do not directly justify immediate large-scale changes to hospital capacity, they highlight the potential efficiency gains achievable through data-driven optimization when integrated into broader planning processes.

## Limitations

This study has several limitations. First, the assumption of uniform travel conditions may not reflect real-world variability such as traffic congestion or road infrastructure disparities. Second, the model considers only travel time by car, not by public transport. Third, the analysis does not consider factors beyond travel time, such as hospital capacity, which are critical for comprehensive access optimization. Incorporating hospital capacity could lead to even more realistic models. In addition, regulatory and organizational factors, such as service mandates, were not explicitly modeled. Consequently, the optimization results should be interpreted as theoretical potential rather than immediately actionable restructuring guidance. Incorporating such system-level constraints in future models could improve the policy relevance of optimization-based planning approaches.

## Conclusion

In conclusion, our study highlights the potential of optimization techniques to enhance healthcare accessibility. *p*-median optimization using linear programming emerged as a useful method for planning purposes on a supra-regional level. Its effectiveness in reducing travel time while improving compliance with governmental accessibility standards was demonstrated. The findings of the study emphasize the potential of optimization methods to enhance access to medical care by restructuring healthcare infrastructure. Future research could explore additional constraints, such as hospital capacity limits, to align the model more closely with real-world conditions.

## Electronic supplementary material

Below is the link to the electronic supplementary material.

Supplementary Material 1

## Data Availability

The datasets used and/or analysed during the current study are available from the corresponding author on reasonable request. For a stepwise explanation of the optimization model, see the supplementary Python script.
